# Coated Mg Alloy Implants: A Spontaneous Wettability Transition Process with Excellent Antibacterial and Osteogenic Functions

**DOI:** 10.3390/ma18091908

**Published:** 2025-04-23

**Authors:** Sijia Yan, Shu Cai, You Zuo, Hang Zhang, Ting Yang, Lei Ling, Huanlin Zhang, Jiaqi Lu, Baichuan He

**Affiliations:** Key Laboratory of Advanced Ceramics and Machining Technology of Ministry of Education, School of Materials Science and Engineering, Tianjin University, Tianjin 300072, China; ysj_@tju.edu.cn (S.Y.); zuoyou_6@tju.edu.cn (Y.Z.); hangzhang123@tju.edu.cn (H.Z.); yangting_@tju.edu.cn (T.Y.); linglei@tju.edu.cn (L.L.); zhanghuanlin@tju.edu.cn (H.Z.); emilylu@tju.edu.cn (J.L.); hbc@tju.edu.cn (B.H.)

**Keywords:** superhydrophobic composite coating, wettability transition, magnesium alloy, antibacterial property, osteogenic property

## Abstract

AZ31B magnesium alloy (wt.%: Al 2.94; Zn 0.87; Mn 0.57; Si 0.0112; Fe 0.0027; Cu 0.0008; Ni 0.0005; Mg remaining) has appropriate mechanical properties, good biodegradability and biocompatibility and can be used as a good orthopedic implant material. AZ31B magnesium alloy with a superhydrophobic surface exhibits excellent corrosion resistance and antibacterial adhesion performance, but superhydrophobic surfaces also hinder osteoblast adhesion and proliferation on the implants, resulting in unsatisfactory osteogenic properties. Therefore, it is necessary to achieve the wettability transition of the superhydrophobic surface at an early stage of implantation. In this work, superhydrophobic hydroxyapatite (HA)/calcium myristate (CaMS)/myristic acid (MA) composite coatings were prepared on AZ31B magnesium alloy using the hydrothermal and immersion methods. The composite coatings can spontaneously undergo the wettability transition from superhydrophobic to hydrophilic after complete exposure to simulated body fluid (SBF, a solution for modeling the composition and concentration of human plasma ions) for 9 h. The wettability transition mainly originated from the deposition and growth of the newly formed CaMS among the HA nanopillars during immersing, which deconstructed the micro-nano structure of the superhydrophobic coatings and directly exposed the HA to the water molecules, thereby significantly altering the wettability of the coatings. Benefiting from the superhydrophobic surface, the composite coating exhibited excellent antibacterial properties. After the wettability transition, the HA/CaMS/MA composite coating exhibited superior osteoblast adhesion performance. This work provides a strategy to enable a superhydrophobic coating to undergo spontaneous wettability transition in SBF, thereby endowing the coated magnesium alloy with a favorable osteogenic property.

## 1. Introduction

AZ31B magnesium alloy is a commercially available material with magnesium as the base and aluminum and zinc as the main alloying elements [1]. It is often used as a structural component in the automotive industry, aerospace field, and electronics industry [2]. In recent years, AZ31B magnesium alloy has shown great potential in orthopedic implants because of its proper mechanical strength and good biocompatibility [3,4,5]. However, severe corrosion and rapid degradation rate of magnesium alloys in a physiological environment can lead to quick loss of mechanical support and local hydrogen accumulation, which limits its clinical application [6,7,8]. Surface modification of magnesium alloys has been confirmed to be one of the most effective methods to solve the above problems [9,10].

Hydroxyapatite (HA) is often used as an ideal coating material because its chemical composition is similar to the main inorganic components of human bones and teeth and has good biocompatibility and osteogenic properties [11,12,13]. Among the methods of preparing HA on the surface of magnesium alloys, the hydrothermal method, a synthetic method that reacts at high temperatures and pressures, has received wide attention because of the advantages of strong bonding, good uniformity, adjustable microstructure, simple operation, and low cost of the prepared coatings [14]. Studies have shown that hydrothermal preparation of HA coatings is one of the effective methods to improve the corrosion resistance and osteogenic properties of magnesium alloys. Sun et al. [15] prepared a hydroxyapatite coating on an AZ31B substrate using a hydrothermal treatment, which exhibited excellent corrosion resistance and osteoblast activity.

Bacterial infection is a common issue in bone repair, which usually leads to implant failure [16,17,18]. Specifically, bacteria can adhere to the implant surface at the early stage of implantation (4–6 h), subsequently forming a biofilm at the implant site [19]. Once a biofilm forms, bacteria growing in the biofilm cannot be eliminated by the immune response of the host and traditional antibacterial treatment [20,21]. Therefore, it is necessary to prevent the formation of biofilm to mitigate implant-related infections.

The construction of bio-inspired superhydrophobic surfaces as a new strategy to improve bacterial adhesion resistance is expected to solve the problem of bacterial film formation on implant surfaces, which has attracted increasing attention [22,23,24]. The superhydrophobic surface is usually achieved by constructing a micro-nano structure and modification with low surface energy substances, and air can be trapped in a nano-scale gap to form an air layer in the solid–liquid interface, where the solid surface cannot be wetted completely [25]. Therefore, the air layer can minimize the contact area between the substrate and the bacteria, thus improving the bacterial adhesion resistance [26,27,28] and enhancing corrosion resistance [29,30,31]. For instance, Ji et al. [26] fabricated a superhydrophobic Quaternized chitosan-based film, which can significantly reduce bacterial adhesion by 95.2%. Zhou et al. [31] designed a series of nickel-coated superhydrophobic surfaces on carbon steel using a one-step electrodeposition route, which, benefited from the presence of an air layer, enabled the samples with a superior corrosion resistance (a −558 mV of E_corr_ and 1.65 × 10^−6^ A·cm^−2^ of I_corr_).

Myristic acid (MA, the molecular formula is C_14_H_28_O_2_) is a kind of straight-chain saturated fatty acid that is widely used in the pharmaceutical and food industries because of its non-toxicity, bioinertness, and biocompatibility [32,33]. Due to the low surface energy of its hydrocarbon chains, MA can be used for surface modification of superhydrophobic surfaces [34,35]. Although the superhydrophobic surfaces can effectively inhibit bacterial adhesion and improve corrosion resistance, they will also hinder the adhesion and growth of osteoblasts. Many studies have shown that hydrophilic surfaces are beneficial for the adhesion and growth of osteoblasts. [36,37]. Therefore, it is desirable to construct a wettability transition coating that can be transformed from superhydrophobic to hydrophilic in the early stage of implantation to simultaneously satisfy the early bacterial adhesion resistance and good osteogenic properties.

Currently, research on wettability transition coatings focuses on the construction of stimulus-responsive materials, which utilize external stimuli such as light, temperature, ultrasound, and magnetism to achieve the switching of two wetting states [38,39,40,41]. In the biomedical field, the wettability transition by external stimulation generally requires stringent stimulation conditions, which can cause no damage to human tissues. For example, Meng et al. [42] achieved a wettability transition for a superhydrophobic hydroxyapatite/bismuth sulfide/lauric acid composite coating (contact angle decreased from 156° to 60.6°) by 5 min of 808 nm near-infrared light irradiation. This method is harmless to human tissues, but 808 nm near-infrared light has limited penetration ability to the human body [43]. Therefore, the optimal approach is to construct superhydrophobic coatings on the surface of magnesium alloy implants that can spontaneously undergo wettability transition in the early stages of implantation in vivo.

**Figure 1:**
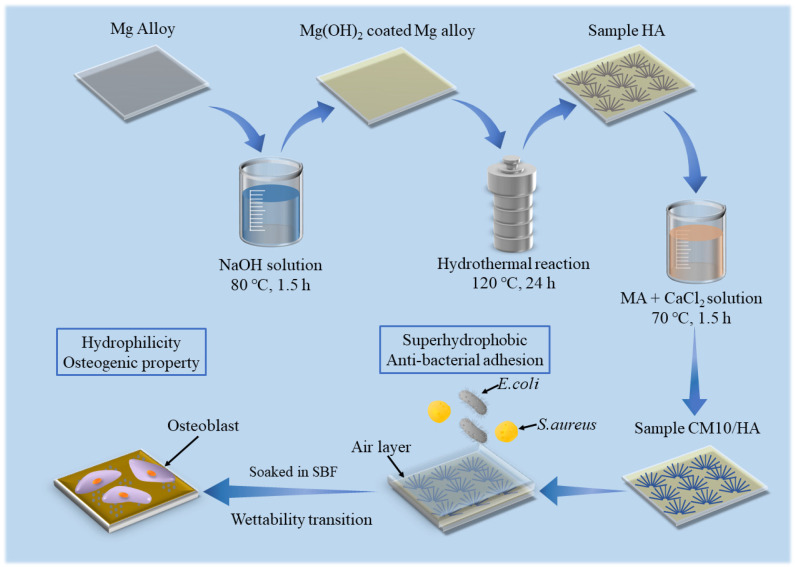
Schematic diagram of work.

In this study, HA coatings with micro-nano structure were prepared on AZ31B magnesium alloys by hydrothermal method, which was followed by an immersion treatment in solution with an optimized proportion of MA and calcium myristate (CaMS) to obtain a superhydrophobic surface with the ability of spontaneous wettability transition. The spontaneous wettability transition ability enabled the coating with a superior antibacterial property before the transition and a good osteogenic property after the transition. The prepared superhydrophobic samples were immersed in simulated body fluid (SBF) to observe the changes in the surface contact angles and explore the corresponding wettability transition mechanism. In addition, the effect of wettability transition on corrosion resistance was also investigated by electrochemical techniques. The schematic illustration of this work is displayed in Figure 1.

## 2. Materials and Methods

### 2.1. Materials Pretreatment and Preparation

The composition of AZ31 magnesium alloy used in this study was 2.94% Al, 0.87% Zn, 0.57% Mn, 0.0112% Si, 0.0027% Fe, 0.0008% Cu, and 0.0005% Ni (wt%), with a balance of Mg, and the alloy was cut into flakes with dimensions of 10 mm × 10 mm × 2 mm. The preparation of HA coatings on AZ31B alloy can be referenced in our previous work [44]. The AZ31B magnesium alloy was polished and then immersed in a 1.5 M NaOH solution. After that, it was hydrothermally treated in a mixed solution of EDTA-2NaCa (0.2 M) and KH_2_PO_4_ (0.2 M) at 120 °C for 24 h, where EDTA-2NaCa functions as a chelating agent, providing calcium for HA, and KH_2_PO_4_ acts as a source of phosphorus for HA. The resulting coated sample was labeled with HA. Subsequently, a CaMS/MA composite coating was applied in a layer over the HA coating, and calcium chloride (CaCl_2_), a component of simulated body fluids that is relatively safe for humans, was selected as the calcium source for generating CaMS. The specific procedure was to dissolve 1.84 g MA and CaCl₂ into 80 mL of ethanol solution (70%), and then the HA samples were immersed in the above solutions at 70 °C for 1.5 h, respectively. The samples immersed in different concentrations of CaCl₂ were labeled as CMn/HA (n represents 5 mM, 10 mM, and 20 mM, respectively). A coated sample without calcium chloride in MA was prepared by the same processes and marked as MA/HA.

### 2.2. Materials Characterization

The microscopic morphology of the coated samples was observed using a cold field emission scanning electron microscope (S-4800, Hitachi, Tokyo, Japan) and a transmission electron microscope (JEM-F200, JEOL, Tokyo, Japan). The surface elemental composition of the coated samples was characterized using an energy dispersive spectroscopy (XFlash7, Bruker, Ettlingen, Germany). The composition of all samples was confirmed by X-ray diffraction (D8 Advance, Bruker, Ettlingen, Germany) in offset mode with 2θ ranging from 10° to 70° at a scan rate of 4 (°)·min^−1^. The chemical functional group of the coatings was analyzed using a Fourier transform infrared spectrometer (Nicolet IS10, Thermo Fisher Scientific, Waltham, MA, USA) in the wavelength range of 500 cm^−1^–4000 cm^−1^. To assess the wettability of the coated samples, the contact angle of the samples was measured using an instrument (DSA 100, Krüss, Hamburg, Germany) at room temperature with a relative humidity of approximately 48% and a droplet volume of 5 μL. Five parallel samples were tested in each group to ensure the reproducibility of the measurements.

### 2.3. Surface Wettability Transition

To study the spontaneous wettability transition of the coatings, the coated samples were immersed in simulated bodily fluids (SBF, the components of the SBF are shown in Table S1 in the Supporting Information) at 37 °C for 6 and 9 h, and the corresponding samples were designated as MA/HA-6 and MA/HA-9, respectively. Similarly, the samples CMn/HA (n = 5, 10, and 20) that were immersed for 6 and 9 h were labeled as CMn/HA-6 and CMn/HA-9.

### 2.4. Electrochemical Measurements

To assess the short-term corrosion resistance of the samples, the electrochemical properties were examined using a CHI660E electrochemical workstation (CHI660E, Shanghai Chenhua, Shanghai, China). Before testing, an epoxy resin coating was applied to the uncoated portion of the samples, with a test area of 1 cm^2^ for the coatings. A three-electrode system was employed, in which the sample, a saturated calomel electrode, and a platinum electrode were used as work electrode, reference electrode, and counter electrode, respectively. The tests were carried out in SBF at 37 °C and started after the sample was immersed for 30 min. Electrochemical impedance spectroscopy (EIS) tests were performed in the frequency range of 0.01–100 kHz, and kinetic potential polarization curve tests were carried out at a scan rate of 1 mV/s in the range of −0.5 V to 0.5 V vs. open circuit potential. Five parallel samples were tested in each group to ensure the reproducibility of the measurements.

### 2.5. Antibacterial Activity

The antibacterial adhesion property of the coated samples was evaluated by the adhesion rates of *Escherichia coli (E. coli)* and *Staphylococcus aureus (S. aureus)* on the surface of the coated samples. The bacteria were cultured and diluted using Luria–Bertani (LB) medium. The samples and 24-well plates were sterilized with UV for 30 min. After that, the samples were introduced into a 24-well plate with diluted bacteria and co-cultured for 4 and 6 h at 37 °C. Subsequently, the live and dead bacteria on the samples were stained, and the bacteria that adhered to the samples were observed under an inverted fluorescence microscope. The equation for the antibacterial adhesion rate (η) of the sample is as follows:

η/% = (1 − S_1_/S_HA_) × 100 (1)

where S_1_ represents the bacterial adhesion area of the superhydrophobic sample MA/HA or CM10/HA, and S_HA_ represents that of the sample HA. S_1_/S_HA_ represents the ratio of bacteria adhering to the surface of superhydrophobic samples relative to sample HA. 1 − S_1_/S_HA_ represents the ratio of non-adherent bacteria on the surface of superhydrophobic samples compared with sample HA, which is used to indicate the antibacterial adhesion rate of the superhydrophobic sample [24,45].

### 2.6. In Vitro Cytocompatibility Assay

The cytotoxicity of the samples was evaluated using mouse osteoblasts (MC3T3-E1, Huaer Bio, Wuhan, China). MC3T3-E1 osteoblasts with a cell density of 1.0 × 10^5^ cells/mL were inoculated into 96-well plates and then cultured in a cell culture incubator at 37 °C for 24 h. The UV-sterilized samples were immersed in 1.25 mL of cell culture medium for 1, 3, and 5 days to obtain the sample extracts. The medium in the 96-well plate that had been inoculated with osteoblasts was removed, and the sample extracts of corresponding days were added and co-cultured with the cells for 1, 3, and 5 days. After each incubation period, a Cell Counting Kit-8 (CCK-8, Solarbio, Beijing, China, for cell proliferation and toxicity assays) solution was added to each well and incubated in the dark for 3 h. The metabolites of living cells were able to reduce WST-8 (the main component of CCK-8) to methyl filth, thus affecting the optical density (OD) value of the solution. The OD value of the solution was then determined using a Microplate Reader (Synergy H1, BioTek, Winooski, VT, USA) at 450 nm.

To observe osteoblast adhesion on the coated samples before and after the wettability transition, the samples were co-cultured with osteoblasts (5 × 10^4^ cells/mL, 6 h) and observed by fluorescence inverted microscopy.

### 2.7. Statistical Analysis

All the data in this article are presented as the mean ± standard deviation. To evaluate the significant differences, an independent samples *t*-test was used for analysis. * *p* < 0.05 and ** *p* < 0.01 are considered to be statistically significant.

## 3. Results and Discussion

### 3.1. Preparation of Superhydrophobic Composite Coatings on Magnesium Alloys

#### 3.1.1. Coating Characterization

**Figure 2:**
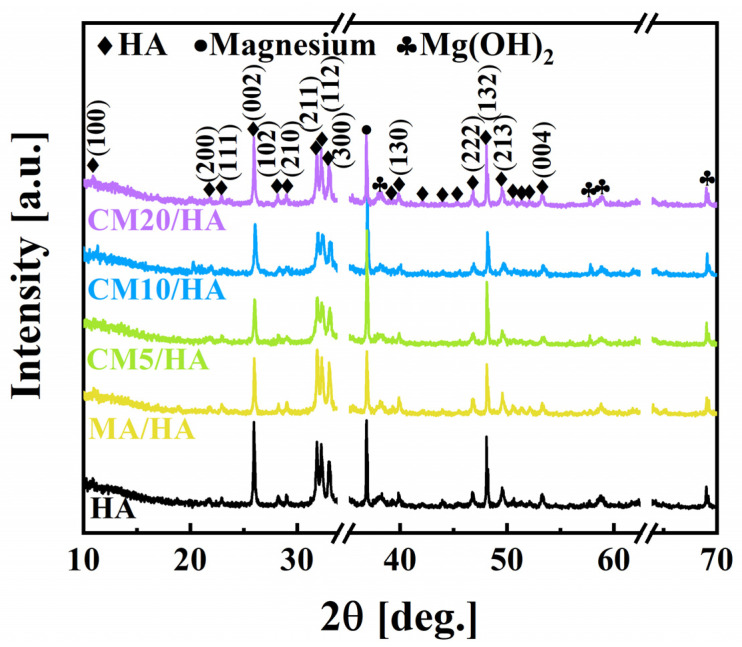
Phase composition of samples immersed in myristic acid fluid with different concentrations of CaCl_2_.

The composite coatings on magnesium alloys were obtained by hydrothermal method and followed immersion treatment in myristic acid solutions with different concentrations of CaCl_2_, of which the XRD patterns are shown in Figure 2. It can be seen that the sample HA prepared by the hydrothermal method contained HA (JCPDS NO. 74-0565), Mg(OH)_2_ (JCPDS NO. 76-0667), and Mg (JCPDS NO. 65-3365), indicating that the HA coating was successfully coated on the surface of the magnesium alloy. Moreover, the XRD results showed that the prepared HA belongs to the hexagonal crystal system with the point group P63/m. After immersion treatment in MA solution or MA containing different concentrations of CaCl_2_, the XRD patterns of the obtained samples exhibited no significant differences from the sample HA, suggesting that different concentrations of CaCl_2_ during the immersing treatment had no affection on the phase composition of the HA coating.

**Figure 3:**
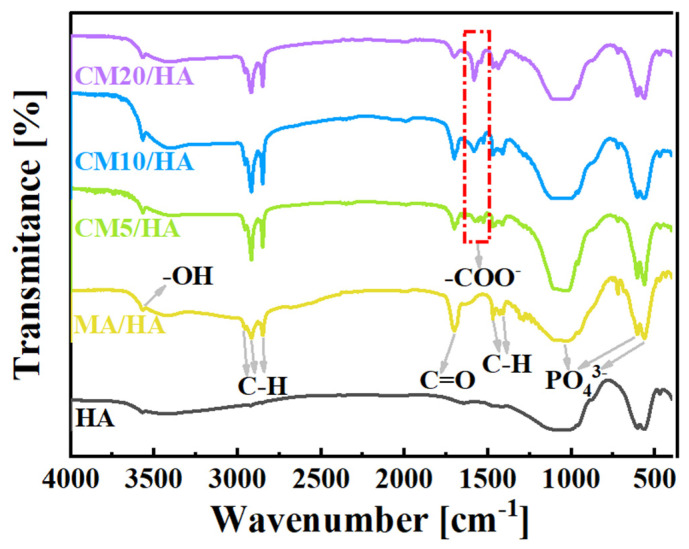
FT-IR spectra of samples immersed in myristic acid solution with different concentrations of CaCl_2_.

To clarify the effect of CaCl_2_ concentration on the coating composition, sample HA and samples after immersing treatment were tested by FT-IR. As illustrated in Figure 3, the peak at 3570 cm^−1^ for the sample HA was attributed to the stretching vibration of -OH, and the characteristic absorption peaks of PO_4_^3−^ can be observed at 560 cm^−1^, 600 cm^−1^, and 1050 cm^−1^. After immersing treatment in MA, in addition to the characteristic peaks of HA, all the characteristic peaks of MA can be observed for sample MA/HA, such as stretching vibrational absorption peak of C=O at 1702 cm^−1^, asymmetric and symmetric stretching vibrational peaks of C-H at 2962 cm^−1^, 2914 cm^−1^, and 2846 cm^−1^ as well as bending vibrational absorption peaks of C-H at 1410 cm^−1^, and 1470 cm^−1^. After adding different concentrations of CaCl_2_ in MA, the absorption peak of -COO- was detected at 1575 cm^−1^ for all samples (CMn/HA, n = 5, 10, and 20), and the peak intensity of the -COO- absorption peak showed a gradual increase with an increase in the CaCl_2_ concentration. Based on the above analysis, it can be inferred that CaMS was generated on the coatings after the introduction of CaCl_2_ into myristic acid.

To further confirm the formation of CaMS on the coatings, the EDS analyses of the coatings with different concentrations of CaCl_2_ were performed, and the results are presented in Figures S1–S5 (Supporting Information). The Ca/P ratio for the coating without CaCl_2_ immersion treatment was 1.68, which was almost identical to that of hydroxyapatite (1.67). When the concentration of CaCl_2_ was up to 20 mM, the Ca/P ratio of sample CM20/HA increased to 1.82. This phenomenon can be attributed to the CaMS formation on the HA coatings, which was consistent with the FT-IR results that -COO- peak was intensified with an increase in the CaCl_2_ concentration.

**Figure 4:**
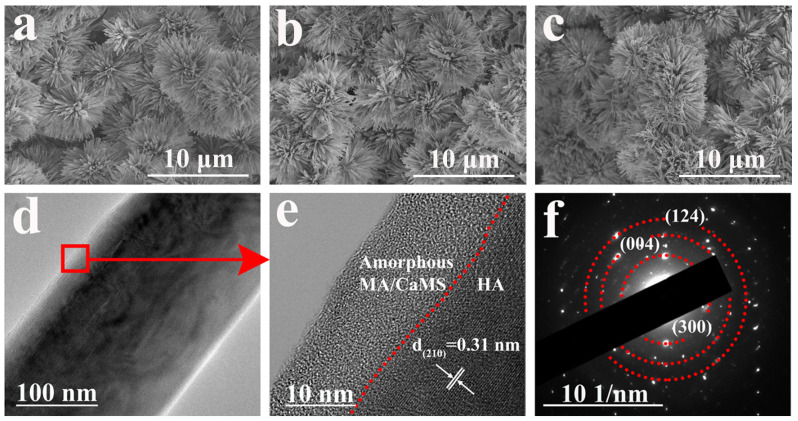
(**a**–**c**) SEM images of HA (**a**), MA/HA (**b**) and CM10/HA (**c**); (**d**,**e**) TEM and HR-TEM images of HA nanopillar of CM10/HA; (**f**) SAED pattern of CM10/HA corresponding to (**d**).

As there was no significant change in the morphology for samples after immersion treatment in myristic acid with different concentrations of CaCl_2_ (Figure S6, Supporting Information), sample CM10/HA was selected as a representative for analysis. Figure 4a–c illustrates the morphological characteristics of samples HA, MA/HA, and CM10/HA, and all samples exhibited a similar morphology, suggesting that the immersion treatment in MA or CaCl_2_/MA had no obvious influence on the microstructure of HA coating (cross-section image is shown in Figure S7, the thickness of CM10/HA coating is around 17 μm). However, no characteristic peaks of CaMS were detected in XRD patterns of sample CM10/HA; it can be inferred that the CaMS was amorphous. Moreover, a TEM analysis was performed to further identify the crystalline state of the CaMS on these composite coatings. As shown in Figure 4d,e, the (210) plane of HA can be observed in sample CM10/HA, and an amorphous nanolayer with a thickness of 10–15 nm existed on the HA nanopillar, which corresponds to the CaMS and MA. The corresponding selected area electron diffraction (SAED) pattern (Figure 4f) shows the (300), (004), and (124) planes of HA, and a halo pattern can be observed, indicating the presence of an amorphous phase, which was consistent with the HR-TEM results (Figure 4e). Based on the above FT-IR and EDS analyses, this amorphous phase was a mixture of MA and CaMS.

#### 3.1.2. Wettability of Composite Coating Surface

**Figure 5:**
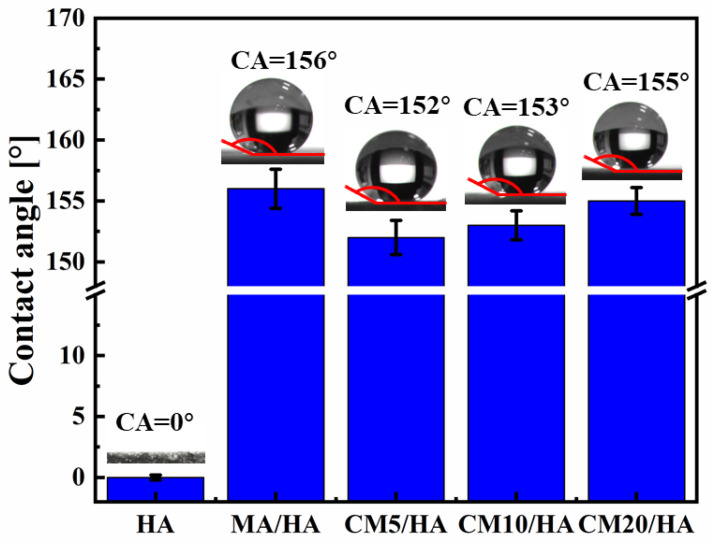
The contact angles from optical images of samples immersed in myristic acid solution with different concentrations of CaCl_2_.

To investigate the effect of the CaCl_2_ concentration on the hydrophobicity of the samples, the contact angles of different samples were tested. When the contact angle is larger than 150°, the surface is confirmed to be superhydrophobic because a contact angle larger than 150° indicates that liquid can barely spread on the surface. As illustrated in Figure 5, the contact angle of sample HA with only micro-nano structure was 0° ± 0.2°. After immersion treatment in MA solution, the contact angle of the sample MA/HA was 156° ± 1.6°, indicating that the sample MA/HA was superhydrophobic. After adding different concentrations of CaCl_2_ to the MA solution, the treated samples (CMn/HA, n = 5, 10, and 20) remained superhydrophobic, with contact angles ranging from 152° to 155°, demonstrating that the addition of CaCl_2_ in the MA solution had little effect on the wettability.

### 3.2. Wettability Spontaneous Transition of Superhydrophobic Composite Coatings in Simulated Body Fluids

**Figure 6:**
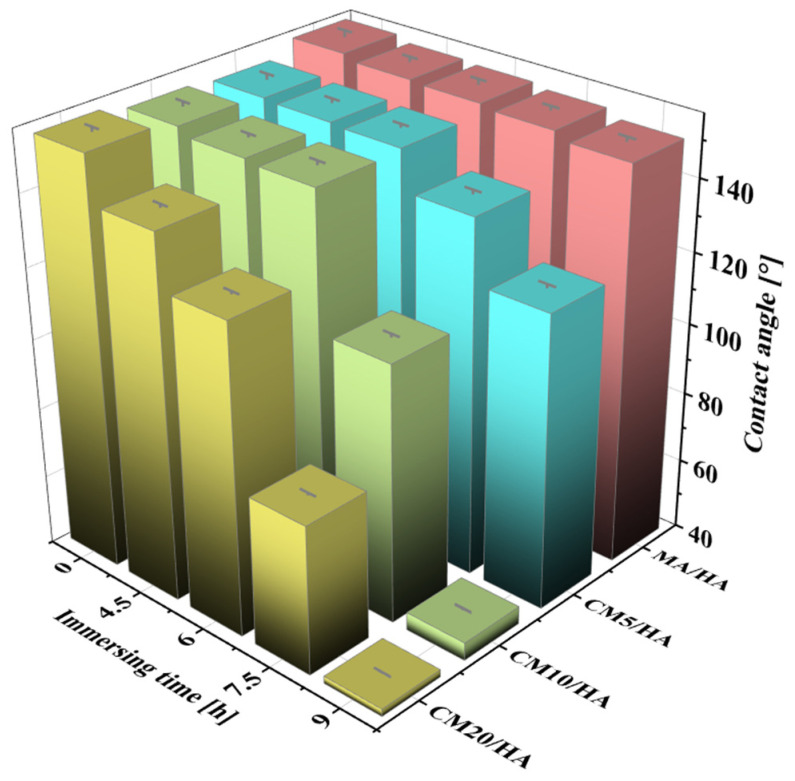
The contact angles of different samples after immersion in SBF for different times.

Magnesium alloy implants with superhydrophobic surfaces have good corrosion resistance and antimicrobial properties, but the superhydrophobic surface hinders the osteoblast adhesion on the bone implants. Therefore, as an implant, it is necessary to achieve wettability transition from superhydrophobic to hydrophilic during the initial implantation stage to enhance its osteogenic properties. The samples MA/HA and CMn/HA (n = 5, 10, and 20) were immersed in SBF at 37 °C for 4.5, 6, 7.5, and 9 h, and the changes in their contact angles are shown in Figure 6. With an increase in immersion time, the contact angles of all samples decreased, but the rates of decrease were significantly different. The contact angle of the sample MA/HA decreased at a slow rate, while that of the sample CMn/HA (n = 5, 10, and 20) with the addition of CaCl_2_ decreased at a relatively fast rate. After immersing for 6 h, the contact angles of the MA/HA, CM5/HA, CM10/HA, and CM20/HA samples were 154° ± 1.3°, 151° ± 0.8°, 150.2° ± 1.0°, and 127° ± 0.6°, respectively. This indicates that the samples MA/HA, CM5/HA, and CM10/HA remained superhydrophobic, and sample CM20/HA was hydrophobic. Due to the fact that bacterial colonization on the surface usually occurs between 4 and 6 h after implantation [46], the sample surfaces must remain superhydrophobic for at least 6 h to ensure good antibacterial adhesion properties. Based on Figure 6, it can be seen that samples MA/HA, CM5/HA, and CM10/HA can maintain excellent antibacterial adhesion performance during this period. However, superhydrophobic surfaces will also inhibit the adhesion and growth of osteoblasts, which is not conducive to osteogenesis [47,48]. Therefore, while ensuring the inhibition of bacterial adhesion, it is necessary to achieve a wettability transition from superhydrophobicity to hydrophilicity to improve its osteogenic performance. Extending the immersing time to 9 h, among the above three samples, only the contact angle of sample CM10/HA decreased to 45.1° ± 0.4°, indicating wettability transition from superhydrophobicity to hydrophilicity, which will be conducive to osteoclast adhesion [49]. According to the changing trend of surface wettability of various samples with immersion time in SBF, it can be inferred that sample CM10/HA was expected to exhibit excellent antibacterial effect during the initial stage of implantation and facilitate osteoblast adhesion and growth after implantation for a short time. Accordingly, the CM10/HA sample is selected for analysis and discussion in the following section.

**Figure 7:**
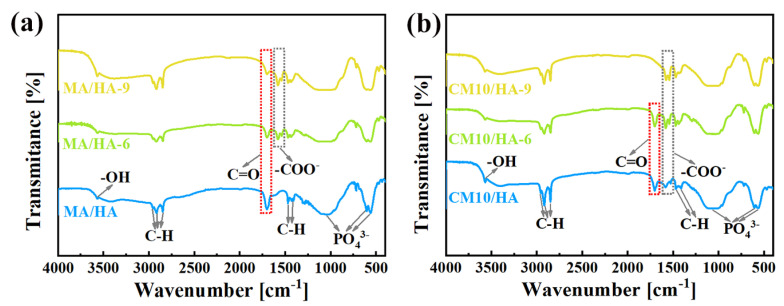
(**a**,**b**) FT-IR spectra of MA/HA (**a**) and CM10/HA (**b**) immersed in SBF for different times.

The wettability transition of coating surfaces is mainly related to the changes in surface composition and microstructure. As illustrated in Figure 7a,b, with an increase in immersion time in SBF, the -COO- absorption peaks of samples MA/HA and CM10/HA gradually intensified, while the C=O peaks weakened. Combined with EDS (Figures S8–S12, Supporting Information), the Ca/P ratios on the surfaces of the two samples also raised gradually, suggesting that newly formed CaMS was deposited on the two samples during the immersion process. Nevertheless, the growth rate of Ca/P on the sample CM10/HA was 0.022 h^−1^, much higher than that of sample MA/HA (0.016 h^−1^), suggesting that the growth rate of CaMS on sample CM10/HA was faster than that of sample MA/HA, which demonstrates that the preformed CaMS on the CM10/HA sample may accelerate the conversion from MA to CaMS during the immersion in SBF, thus changing the composition and microstructure of the coating surface. Although the conversion from MA to CaMS resulted in the replacement of H in the carboxyl group with Ca, the hydrophobic alkyl groups that dominated the molecular nonpolarity of MA were not changed. Thus, this composition change can hardly influence the molecular nonpolarity of the MA and surface energy of the coating. Therefore, it can be concluded that the wettability transition was mainly dominated by the microstructure evolution during immersion in SBF.

**Figure 8:**
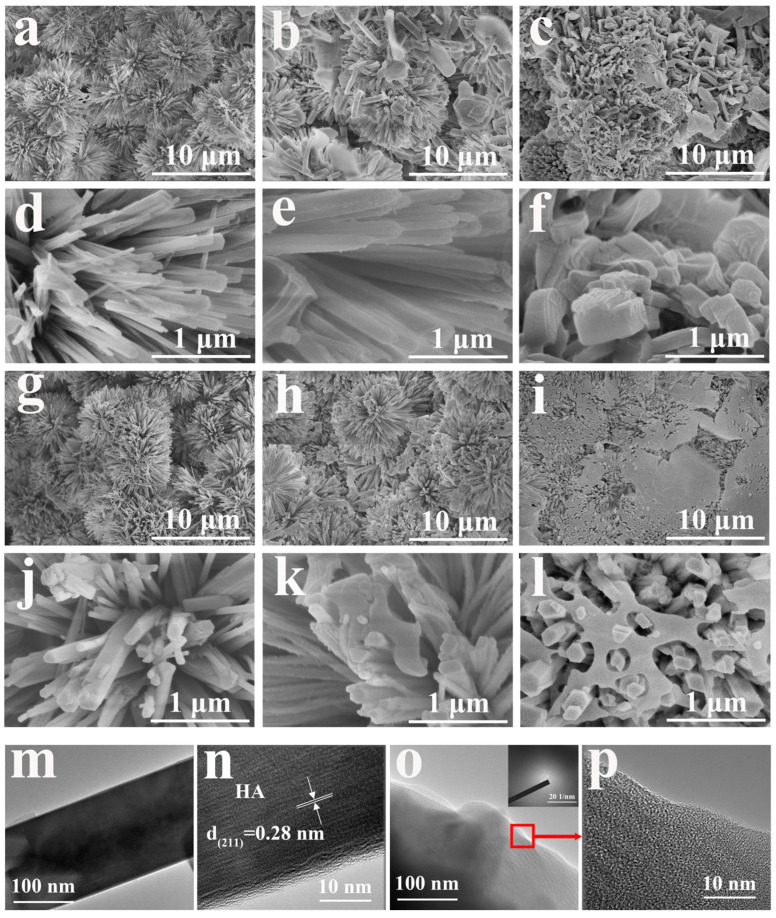
(**a**–**f**) SEM of MA/HA immersed in SBF for different times: (**a**,**d**) 0 h, (**b**,**e**) 6 h, (**c**,**f**) 9 h; (**g**–**l**) SEM of CM10/HA immersed in SBF for different time: (**g**,**j**) 0 h, (**h**,**k**) 6 h, (**i**,**l**) 9 h; (**m**,**n**) TEM and HRTEM images of HA nanopillar of CM10/HA immersed in SBF for 9 h; (**o**,**p**) TEM, HRTEM, and SAED images of amorphous phase of CM10/HA immersed in SBF for 9 h.

As shown in Figure 8a–l, the microstructure of samples MA/HA and CM10/HA showed obvious changes. After soaking for 6 h, there was a small amount of nanoflakes formed on the surface of the MA/HA and CM10/HA but still maintained the original morphology. When immersed for 9 h, the surface morphology of samples MA/HA and CM10/HA underwent significant changes. Figure 8f shows that the stacked CaMS flakes generated on the HA nanopillars of sample MA/HA during the immersion process resulted in the formation of another rough micro-nano structure and could maintain the superhydrophobic characteristic even though the morphology had been changed. For sample CM10/HA (Figure 8i,l), the surface appeared relatively flat, and there was some substance filled around the HA nanopillars, which can be explained by the newly formed CaMS filling among the HA nanopillars. By comparison, it can be found that the preformed CaMS on the sample CM10/HA can provide a large number of nucleation sites for newly formed CaMS during immersion in SBF, promoting the growth of CaMS among the HA nanopillars, thus changing the surface morphology to a smooth plane after 9 h immersion. This change in surface morphology for sample CM10/HA deconstructed the intrinsic micro-nano structure of the HA coating, which erased the prerequisite of the superhydrophobic surface construction and was detrimental to the formation of the air layer in the solid–liquid interface, thus being responsible for the wettability transition. TEM was tested for sample CM10/HA to study the morphology changes and the corresponding reaction mechanism. By comparing Figure 4e and Figure 8n, it is found that the amorphous nanolayer on HA nanopillar almost disappeared after 9 h of immersion, which indicates that MA in the CaMS/MA nanolayer was consumed to form the new CaMS. In the meantime, amorphous CaMS in the nanolayer can serve as nucleation sites for newly formed CaMS, promoting the growth of CaMS and forming a continuous morphology, as shown in Figure 8l. In addition, the disappearance of the CaMS/MA nanolayer led to the direct exposure of hydrophilic HA, which also facilitated the wettability transition of the sample CM10/HA. Moreover, the TEM and SAED (Figure 8o,p) images confirmed that the newly formed CaMS was in an amorphous state.

**Figure 9:**
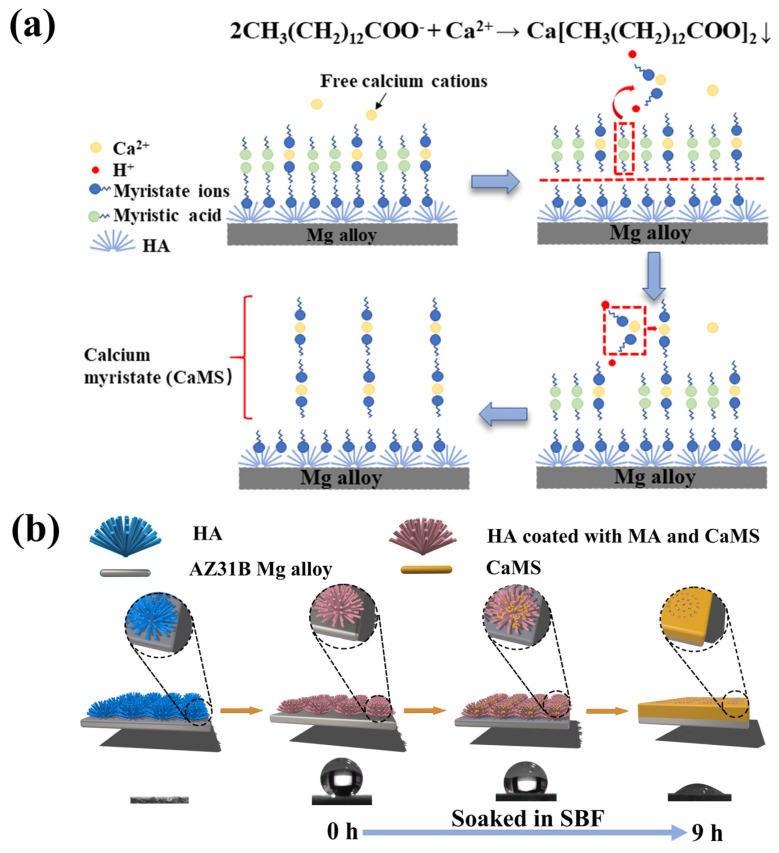
Chemical reactions (**a**) and schematic diagrams (**b**) of the wettability transition process in simulated body fluids.

From the above analysis, the superhydrophobic coating of sample CM10/HA showed a micro-nano structure and low surface energy endowed by the CaMS/MA nanolayer. During the immersion in SBF, the wettability transition process can be illustrated in Figure 9. First, CaMS formed by the reaction of MA with Ca^2+^ from SBF and subsequently deposited and grew among the HA nanopillars of the coating, thereby changing the microstructure of the coating. Meanwhile, with the continuous formation of CaMS, the micro-nano structure was transformed to a relatively smooth surface, making it difficult to create an air barrier layer in the solid–liquid interface. In addition, the consumption of MA in the amorphous CaMS/MA nanolayer resulted in the direct exposure of the hydrophilic HA nanopillars to SBF solution. Therefore, the destruction of the micro-nano structure and the direct exposure of the HA nanopillars synergistically led to the coating wettability transition from superhydrophobic to hydrophilic.

### 3.3. The Influence of Wettability Transition on Corrosion Resistance, Antibacterial and Osteogenic Properties of Composite Coating

The changes in surface wettability of the composite coatings will lead to variations in the coating composition, functional groups, charges, structure, surface energy, etc., which may affect the properties of the composite coatings, such as corrosion resistance, antibacterial ability, and cell adhesion and growth.

#### 3.3.1. The Influence of Wettability Transition on Corrosion Resistance of Composite Coating

**Figure 10:**
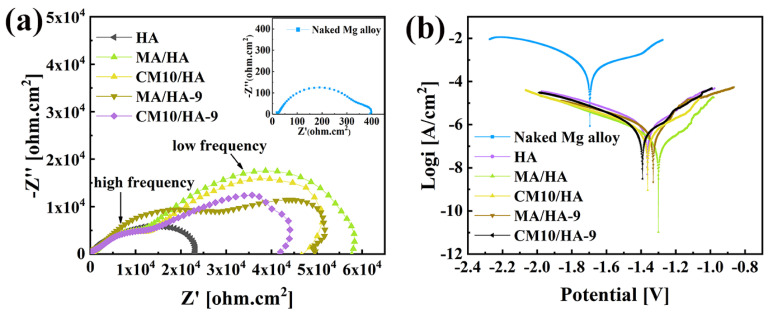
Electrochemical test results of different samples in SBF: (**a**) EIS curves; (**b**) potentiodynamic polarization curves.

Magnesium alloys are susceptible to corrosion and have high degradation rates in human physiological environments [49]. Thus, improvement of the corrosion resistance for magnesium alloys is crucial for its biomedical applications. To evaluate the short-term corrosion resistance of the coatings, electrochemical tests were performed on samples HA, MA/HA, MA/HA-9, CM10/HA, and CM10/HA-9. Figure 10a shows the EIS curve; the size of the capacitance loop diameter can reflect the relative value of the impedance for the samples. The larger the impedance, the lower the corrosion rate. The bare magnesium alloy had the smallest capacitance loop diameter, and the impedance was only 0.33 ± 0.02 kΩ·cm^2^. The impedance of the HA sample was 23.12 ± 1.02 kΩ·cm^2^, which had a significant improvement compared with that of the bare magnesium alloy. The superhydrophobic samples included MA/HA, MA/HA-9, and CM10/HA, and their corresponding impedance values were 57.72 ± 4.52 kΩ·cm^2^, 49.64 ± 3.28 kΩ·cm^2^, and 46.63 ± 2.34 kΩ·cm^2^, respectively, which were 2–3 times higher than that of HA sample, indicating that the superhydrophobic surface can hinder the diffusion of corrosive ions and reduce the corrosion rate. For sample CM10/HA-9 with a hydrophilic surface, even though the impedance value was reduced compared with that of the superhydrophobic sample, it still reached 42.01 ± 2.16 kΩ·cm^2^. In comparison with other biomedical coatings on Mg alloys which have been published previously (Figure S13), the sample CM10/HA-9 still exhibited a decent corrosion resistance (R_p_ value) [50,51,52,53,54]. The good corrosion resistance was due to the generation of the CaMS layer that had a certain degree of resistance to corrosive ions. In addition, the polarization curves of the samples were analyzed, as shown in Figure 10b, the polarization parameter corrosion current density icorr can be extrapolated from the Tafel curve, and the icorr of the samples were sorted as follows: Mg (348.100 ± 4.820 μA/cm^2^) > HA (0.917 ± 0.032 μA/cm^2^) > CM10/HA-9 (0.505 ± 0.026 μA/cm^2^) > CM10/HA (0.396 ± 0.018 μA/cm^2^) > MA/HA-9 (0.328 ± 0.047 μA/cm^2^) > MA/HA (0.147 ± 0.005 μA/cm^2^). The smaller the icorr value, the smaller the corrosion rate and the result agreed with the EIS curve. The above results show that the sample CM10/HA still had a decent protective effect on magnesium alloy both before and after the wettability transition. The parameters from the EIS and potentiodynamic curves are displayed in Table S2. To interpret the reaction mechanism, the EIS results were further analyzed [50,51,52,53,54]. As shown in Figure 10, the EIS curves can be divided into impedance semicircles at high frequency and low frequency. For MA/HA and MA/HA-9, it can be observed that after immersion for 9 h, the impedance semicircles at high frequency and low frequency are larger and smaller (including Z′ and -Z″ directions), respectively, indicating an increase in resistance and capacitance for the top layer of the coating and a decrease in resistance and capacitance for the bottom layer of the coating. This phenomenon represented the micro-nano structural CaMS layer formation on the top layer of HA coating during the immersion process, which transformed the superhydrophobic surface from the surroundings to the tops of the HA nanopillars. According to EIS data for CM10/HA and CM10/HA-9, the 9 h immersion in SBF can only reduce the size of the impedance semicircle at low frequency, corresponding to the deconstruction of micro-nano structure and exposure of HA nanopillars, by which the corrosive ions can facially penetrate the HA coating without an air layer on the interface. In the meantime, the hardly influenced impedance semicircle at high frequency can be attributed to the newly formed CaMS surrounding the HA nanopillars. In addition, we evaluated the long-term corrosion resistance of the coatings by observing the pH values and corrosion rates of the samples after immersing them in SBF for a different number of days (Figure S14). The pH of sample CM10/HA fluctuated between 7.4 and 7.73 during seven days of immersion in SBF, and the corrosion rate was never higher than 0.41 mg·cm^−2^·day^−1^, indicating good corrosion resistance.

#### 3.3.2. The Influence of Wettability Transition on Antibacterial Property of Composite Coating

**Figure 11:**
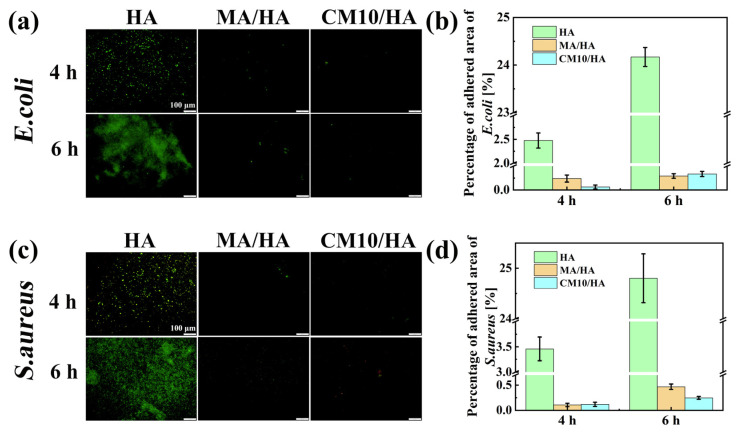
Fluorescence images and fluorescence area percentages of HA, MA/HA, and CM10/HA after co-culturing with different bacterial solutions for 4 and 6 h: (**a**,**b**) *E. coli*; (**c**,**d**) *S. aureus*.

To assess the antibacterial adhesion property of the composite coating at its initial stage, the samples HA, MA/HA, and CM10/HA were co-cultured with *E. coli* and *S. aureus* for 4 and 6 h. The bacteria adhesion to the sample surface was observed by fluorescence staining. As illustrated in Figure 11, when co-cultured with *E. coli* for 4 h and 6 h, the antibacterial adhesion rates of the MA/HA and CM10/HA were 90.4% and 97.5%, as well as 98.8% and 98.6%, respectively. When co-cultured with *S. aureus* for 4 h and 6 h, the antibacterial adhesion rates of the two samples were 96.9% and 96.6% as well as 98.1% and 99.0%, respectively, suggesting that both surfaces had excellent antibacterial adhesion properties against *E. coli* and *S. aureus*, and were far superior to sample HA. This phenomenon can be attributed to the hydrophilic character of sample HA, which provides a larger surface area for contact with the bacterial solution, thereby increasing the possibility of bacterial adhesion to the surface. However, an air layer is present between the superhydrophobic surfaces of samples MA/HA and CM10/HA and the bacterial liquid. This air layer prevents direct contact between the bacteria and the sample surfaces, thereby reducing the probability of bacterial adhesion to the sample surfaces.

#### 3.3.3. The Influence of Wettability Transition on Osteogenic Property of Composite Coating

**Figure 12:**
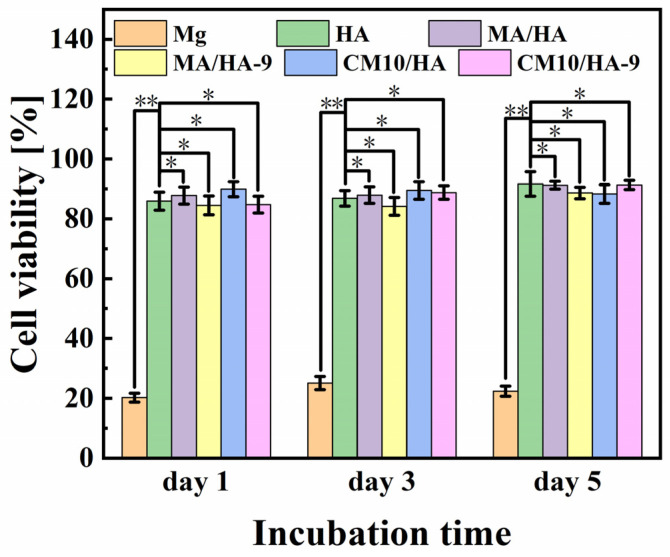
Cell viability of MC3T3-E1 osteoblasts incubated with different samples for 1, 3, and 5 days (* *p* < 0.05, ** *p* < 0.01).

The cell viability of MC3T3-E1 osteoblasts incubated with different samples for 1, 3, and 5 days is shown in Figure 12. For bare Mg alloy, the cell viability is poor, ranging from 20% to 30%, which was mainly due to the apoptosis and the cell death caused by a strong alkaline environment from the rapid degradation of Mg. In contrast, HA, MA/HA, MA/HA-9, CM10/HA, and CM10/HA-9 samples showed good cell activity after co-culture with the cells, and the cell survival rates were higher than 84%, indicating the noncytotoxicity of all samples.

**Figure 13:**
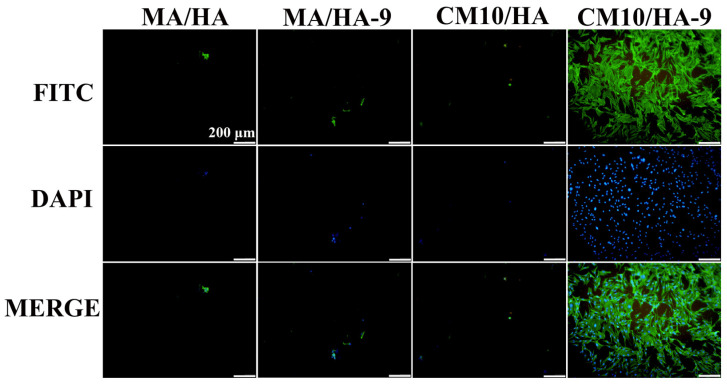
Fluorescence images of MC3T3-E1 osteoblasts adhering to different sample surfaces (the green color represents the actin in the cells, and the blue color represents the cell nucleus).

The surface wettability of biomaterials plays an important role in the adhesion of osteoblasts. Studies have shown that osteoblast adhesion to hydrophilic surfaces is more facile [55]. To clarify the effect of the wettability transition of the superhydrophobic surfaces on the osteoblastic activity, samples MA/HA, MA/HA-9, CM10/HA, and CM10/HA-9 were co-cultured with osteoblasts for 6 h, and the adhesion of osteoblasts on the sample was observed by fluorescence staining. As shown in Figure 13, the number of osteoblasts adhered to the surface of superhydrophobic samples MA/HA, MA/HA-9, and CM10/HA was very small, and the morphology of the cells was not the regular shuttle shape, which may be due to the superhydrophobic characteristic of the surface. On the contrary, the number of osteoblasts adhered to the surface of the CM10/HA-9 sample in a hydrophilic state was increased significantly, and the cells exhibited a shuttle shape, indicating that the wettability transition of the coating from superhydrophobicity to hydrophilicity was beneficial for the adhesion and growth of osteoblasts.

## 4. Conclusions

1. A superhydrophobic composite coating of CaMS-MA/HA was prepared on magnesium alloys using a hydrothermal method and followed by an immersion treatment in myristic acid solutions with different CaCl_2_ concentrations, with contact angles ranging from 152° to 155°.
2. The optimized superhydrophobic composite coating on magnesium alloy had excellent corrosion resistance with high impedance (46.63 ± 2.34 kΩ·cm^2^) and low corrosion current density (0.396 ± 0.018 μA/cm^2^), and the anti-adhesion rates of both *E. coli* and *S. aureus* were more than 90%, indicating the effective inhibition of bacterial adhesion and colonization on the implant surface.
3. In SBF, the superhydrophobicity of the coating can be spontaneously transitioned into hydrophilicity, and the contact angle decreases to 45.1° ± 0.4° after immersing for 9 h. The coated magnesium alloy had good osteogenic properties after the wettability transition and still maintained a decent corrosion resistance.
4. The spontaneous wettability transition of the coating surface in SBF mainly originated from the synergistic effect of deconstruction of micro-nano structure and exposure of HA nanopillars.

## Data Availability

The original contributions presented in this study are included in the article and Supplementary Materials. Further inquiries can be directed to the corresponding author.

## Supplementary Materials

The following supporting information can be downloaded at: https://www.mdpi.com/article/10.3390/ma18091908/s1, Figure S1: EDS analysis of the MA/HA sample; Figure S2: EDS analysis of the CM5/HA sample; Figure S3: EDS analysis of the CM10/HA sample; Figure S4: EDS analysis of the CM20/HA sample; Figure S5: Calcium-phosphorus ratios of samples immersed in myristic acid fluid with different concentrations of CaCl_2_; Figure S6: SEM images of CM5/HA (a), CM10/HA (b) and CM20/HA (c); Figure S7: The cross-section image of CM10/HA coating on Mg alloy; Figure S8: EDS analysis of the MA/HA-6 sample; Figure S9: EDS analysis of the MA/HA-9 sample; Figure S10: EDS analysis of the CM10/HA-6 sample; Figure S11: EDS analysis of the CM10/HA-9 sample; Figure S12: Calcium-phosphorus ratios of MA/HA and CM10/HA immersed in SBF for different time; Figure. S13: The Rp values of coatings in this work in comparison with other biomedical coatings on Mg alloys; Figure S14: The pH values (a) and corrosion rates (b) of different samples after being immersed in SBF for different days; Table S1: Reagents used in the preparation of simulated body fluid (SBF) and the corresponding concentrations; Table S2. Parameters for EIS and potentiodynamic curves.

## Abbreviations

The following abbreviations are used in this manuscript:

HA	Hydroxyapatite.
MA	Myristic acid.
CaMS	Calcium myristate.
Sample HA	The magnesium alloys coated with hydroxyapatite coating.
Sample MA/HA	The magnesium alloys coated with a composite coating obtained by immersing the sample HA in myristic acid.
Sample MA/HA-6	The sample MA/HA immersed in simulated body fluid for 6 h.
Sample CMn/HA (n = 5, 10, and 20)	The magnesium alloys coated with a composite coating obtained by immersing the sample HA in myristic acid with different concentrations of CaCl_2_. The n represents the concentration value with a unit of mM.
Sample CMn/HA-6 (n = 5, 10, and 20)	The sample CMn/HA (n = 5, 10, and 20) immersed in simulated body fluid for 6 h.
Sample CMn/HA-9 (n = 5, 10, and 20)	The sample CMn/HA (n = 5, 10, and 20) immersed in simulated body fluid for 9 h.
