# Supplementary material for: Coated Mg Alloy Implants: A Spontaneous Wettability Transition Process with Excellent Antibacterial and Osteogenic Functions
Source: Materials (Basel). 2025 Apr 23;18(9):1908. doi: 10.3390/ma18091908 (PMC12072351; doi:10.3390/ma18091908)
Supplement: Supplementary file 1 [file materials-18-01908-s001.zip › materials-3581876-supplementary.pdf]

Supporting Information

A spontaneous wettability transition of superhydrophobic coating endows Mg alloy implant with excellent antibacterial and osteogenic properties

Sijia Yan[a], Shu Cai[a,*], You Zuo[a], Hang Zhang[a], Ting Yang[a], Lei Ling[a], Huanlin Zhang[a], Jiaqi Lu[a], Baichuan He[a]

[a] *Key Laboratory of Advanced Ceramics and Machining Technology of Ministry of Education, School of Materials Science and Engineering, Tianjin University, Tianjin, 300072, China.*

1. Abbreviations corresponding to substances or samples

1) **HA**: Hydroxyapatite.
2) **MA**: Myristic acid.
3) **CaMS**: Calcium myristate.
4) **Sample HA**: The magnesium alloys coated with hydroxyapatite coating.
5) **Sample MA/HA**: The magnesium alloys coated with a composite coating obtained by immersing the sample HA in myristic acid.
6) **Sample CMn/HA (n=5, 10, 20)**: The magnesium alloys coated with a composite coating obtained by immersing the sample HA in myristic acid with different concentrations of $CaCl_2$. The n represents the concentration value with a unit of mM.
7) **Sample MA/HA-6**: The sample MA/HA immersed in simulated body fluid for 6 hours.
8) **Sample MA/HA-9**: The sample MA/HA immersed in simulated body fluid for 9

hours.

9) **Sample CMn/HA-6 (n=5, 10, 20)**: The sample CMn/HA (n=5, 10, 20) immersed in simulated body fluid for 6 hours.

10) **Sample CMn/HA-9 (n=5, 10, 20)**: The sample CMn/HA (n=5, 10, 20) immersed in simulated body fluid for 9 hours.

2. SEM and EDS test results

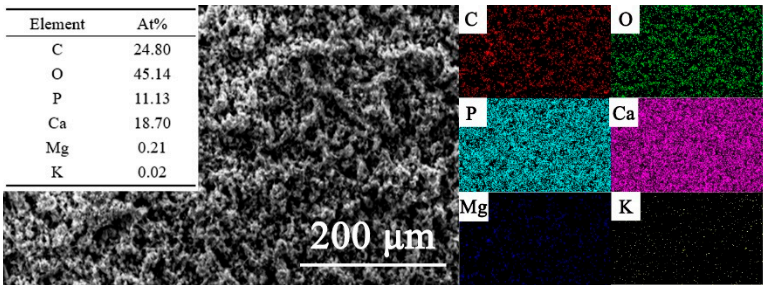

Figure S1. EDS analysis of the MA/HA sample.

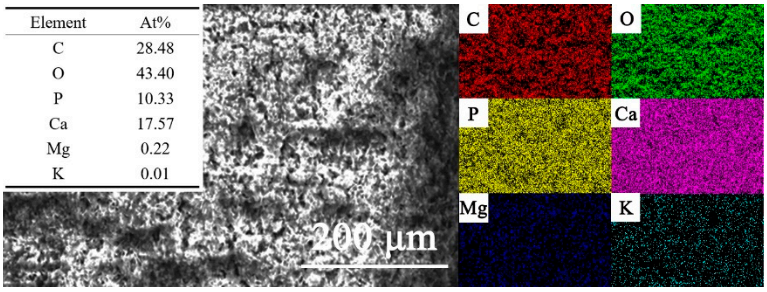

Figure S2. EDS analysis of the CM5/HA sample.

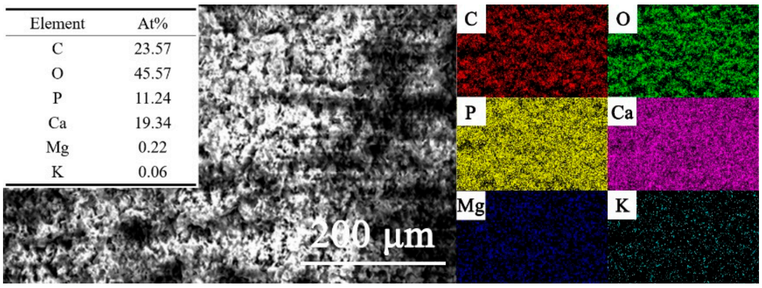

Figure S3. EDS analysis of the CM10/HA sample.

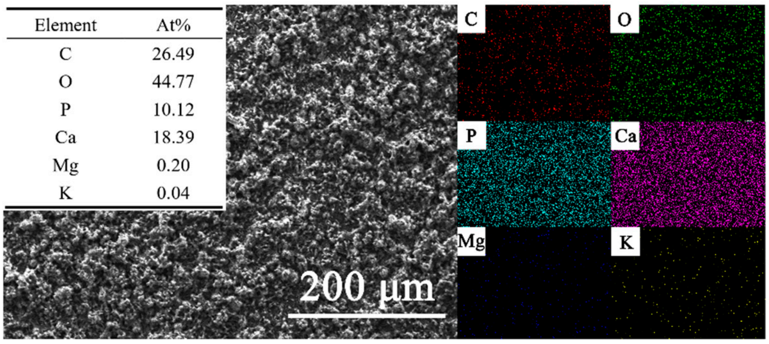

Figure S4. EDS analysis of the CM20/HA sample.

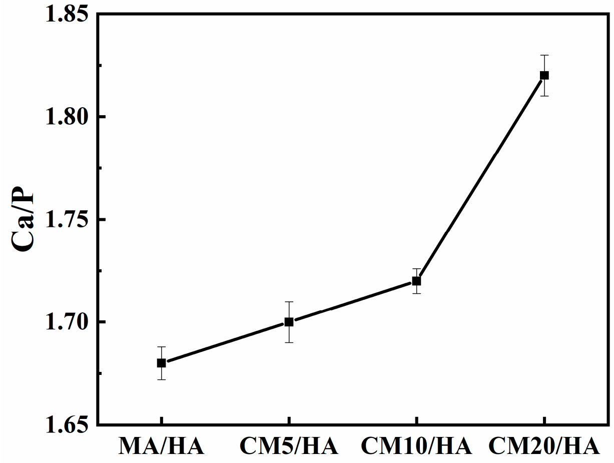

Figure S5. Calcium-phosphorus ratios of samples immersed in myristic acid fluid with different concentrations of $CaCl_2$.

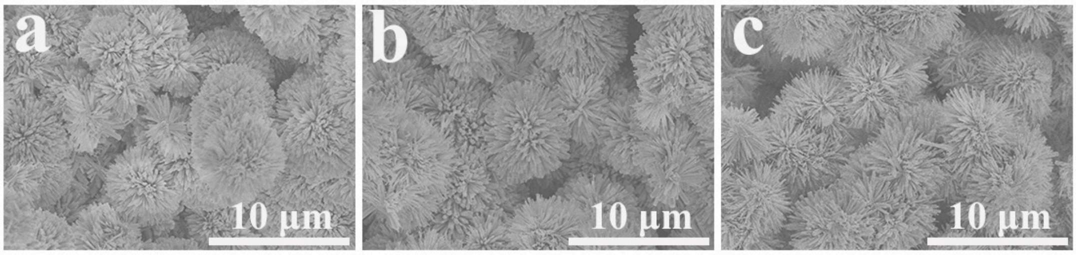

Figure S6. SEM images of CM5/HA (a), CM10/HA (b) and CM20/HA (c)

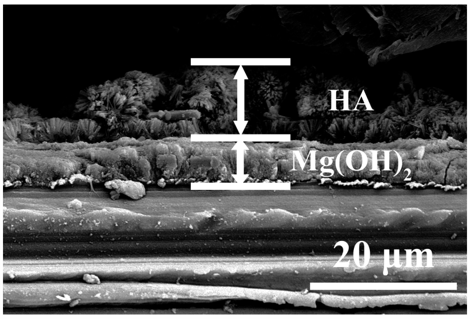

Figure S7. The cross-section picture of CM10/HA coating on Mg alloy.

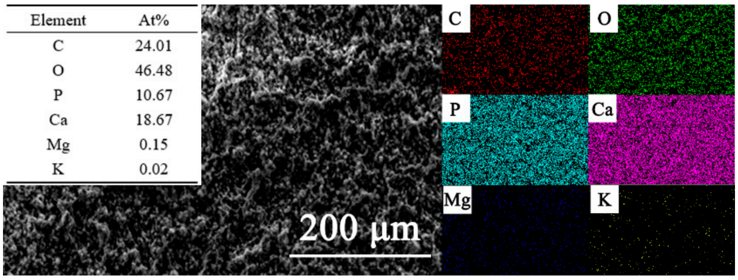

Element	At%
C	24.01
O	46.48
P	10.67
Ca	18.67
Mg	0.15
K	0.02

Figure S8. EDS analysis of the MA/HA-6 sample.

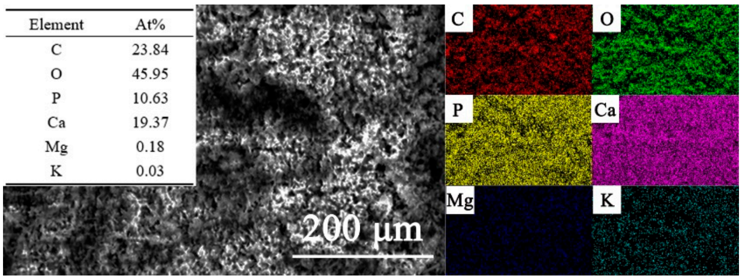

Element	At%
C	23.84
O	45.95
P	10.63
Ca	19.37
Mg	0.18
K	0.03

Figure S9. EDS analysis of the MA/HA-9 sample.

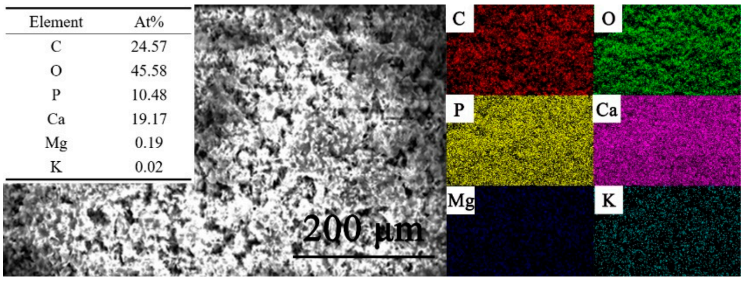

Element	At%
C	24.57
O	45.58
P	10.48
Ca	19.17
Mg	0.19
K	0.02

Figure S10. EDS analysis of the CM10/HA-6 sample.

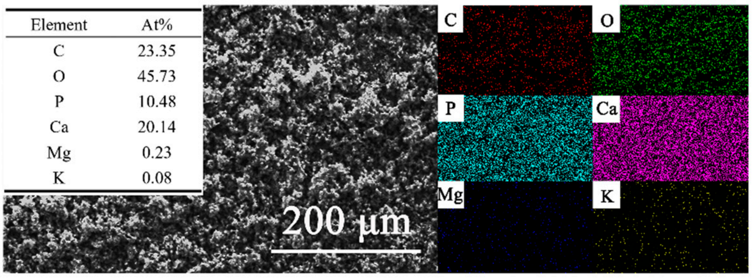

Figure S11. EDS analysis of the CM10/HA-9 sample.

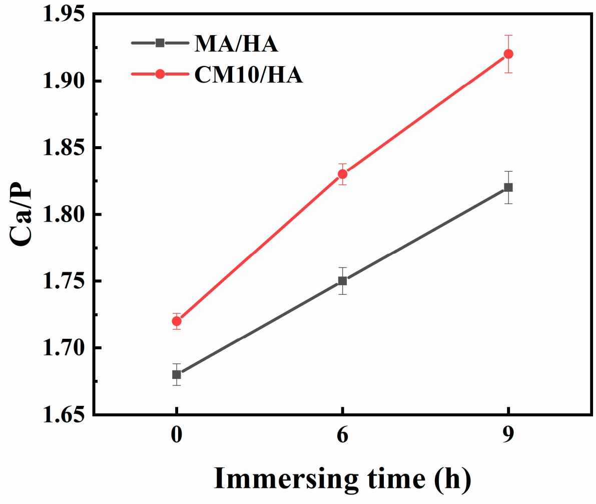

Figure S12. Calcium-phosphorus ratios of MA/HA and CM10/HA immersed in SBF for different time.

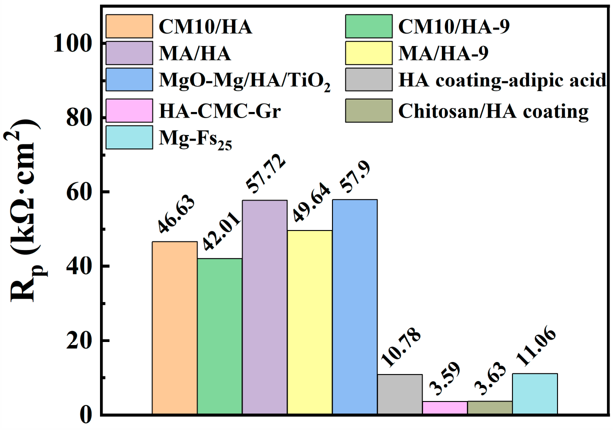

Figure S13. The R_p values of coatings in this work in comparison with other biomedical coatings on Mg alloys.

Table S1. Reagents used in the preparation of simulated body fluid (SBF) and the corresponding concentrations

Reagents	Concentration (mmol/L)
NaCl	112.00
$NaHCO_3$	27.00
$Na_2HPO_4\cdot 12H_2O$	1.00
KCl	5.00
$CaCl_2$	2.50
$MgCl_2\cdot 6H_2O$	1.50
Na_2SO_4	0.50
Tris	16.51

Table S2. Parameters for EIS and potentiodynamic curves

Samples	R_p (kΩ·cm^2)	i_{corr} (μA/cm^2)
Naked Mg alloy	0.33 ± 0.02	348.100 ± 4.820
HA	23.12 ± 1.02	0.917 ± 0.032
MA/HA	57.72 ± 4.52	0.147 ± 0.005
MA/HA-9	49.64 ± 3.28	0.328 ± 0.047
CM10/HA	46.63 ± 2.34	0.396 ± 0.018
CM10/HA-9	42.01 ± 2.16	0.505 ± 0.026

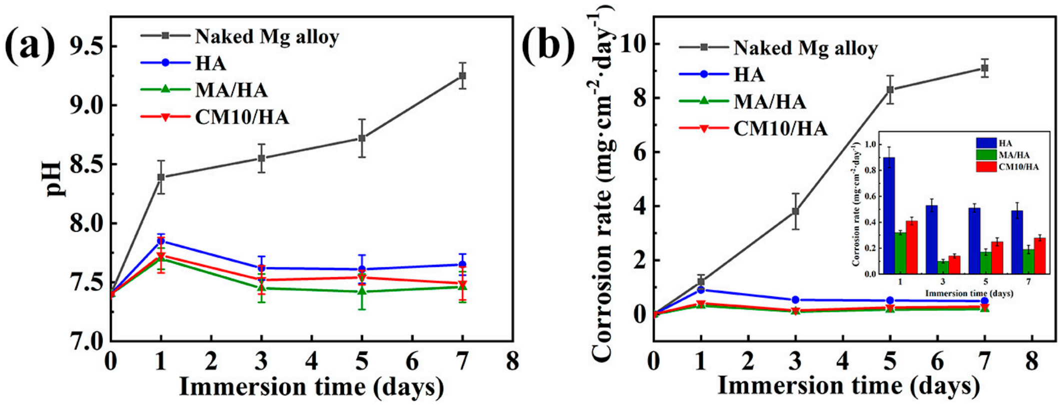

Figure S14. The pH values (a) and corrosion rates (b) of different samples after being immersed in SBF for different days.
